# The neglected nano-specific toxicity of ZnO nanoparticles in the yeast *Saccharomyces cerevisiae*

**DOI:** 10.1038/srep24839

**Published:** 2016-04-20

**Authors:** Weicheng Zhang, Shaopan Bao, Tao Fang

**Affiliations:** 1 Institute of Hydrobiology, Chinese Academy of Sciences, Wuhan, 430072, China; 2 Graduate University of Chinese Academy of Sciences, Beijing 100049, China

## Abstract

Nanoparticles (NPs) with unique physicochemical properties induce nano-specific (excess) toxicity in organisms compared with their bulk counterparts. Evaluation and consideration of nano-specific toxicity are meaningful for the safe design and environmental risk assessment of NPs. However, ZnO NPs have been reported to lack excess toxicity for diverse organisms. In the present study, the nano-specific toxicity of ZnO NPs was evaluated in the yeast *Saccharomyces cerevisiae*. Nano-specific toxicity of ZnO NPs was not observed in the wild type yeast. However, the ZnO NPs induced very similar nano-specific toxicities in the three mutants with comparable log *T*_*e*_^(particle)^ values (0.64 vs 0.65 vs 0.62), suggesting that the mutants were more sensitive and specific for the NPs’ nano-specific toxicity. The toxic effects in the yeast were slightly attributable to dissolved zinc ions from the ZnO (nano or bulk) particles. Oxidative damage and mechanical damage contributed to the toxic effect of the ZnO particles. The mechanism of mechanical damage is proposed to be an inherent characteristic underlying the nano-specific toxicity in the mutants. The log *T*_*e*_^(particle)^ was a useful parameter for evaluation of NPs nano-specific toxicity, whereas log *T*_*e*_^(ion)^ efficiently determined the NPs toxicity associated with released ions.

Engineered nanomaterials (ENMs) with unique and novel physicochemical properties provide not only a lot of benefits but also serious threats once they enter into the environment. Theoretically, the special properties of ENMs can elicit nano-specific (excess) toxicity in organisms compared with their bulk counterparts^1^,^2^,^3^. Due to the potentially excess hazardous effect, the evaluation of the health safety and environmental risk of ENMs is becoming important and urgent. Fortunately, scientists have made great efforts in the safe design and risk assessment of ENMs. For instance, high content screening (HCS) had been successfully utilized to evaluate the environmental and toxicological impacts of NPs^4^. Although the biological and toxic mechanisms that induce toxicity in organisms have been extensively investigated^5^,^6^, nano-specific toxicities have been neglected in health risk assessments for various reasons. A large number of investigations confirmed the existence of nano-specific toxicities. For example, CuO nanoparticles (NPs) showed approximately 50-fold higher toxicity in *Vibrio fischeri, Daphnia magna* and *Thamnocephalus platyurus* than bulk CuO, and TiO_2_ NPs were shown to be toxic in *Daphnia magna* and zebrafish even though bulk TiO_2_ was not toxic^3^,^7^. A comparison of the toxicity of NPs and bulk particles suggested that a novel risk assessment pattern for NPs should be established, because the nano-specific effects are dramatically important and need to be seriously considered in the environmental assessment.

Interestingly, ZnO NPs exerted toxic effects analogous to ZnO bulk particles in various organisms, including bacteria (30 min EC_50_ in *Vibrio fischeri*, 1.9 vs 1.8 vs mg/L)^3^, algae (72 h EC_50_ in *Pseudokirchneriella subcapitata*, 0.042 vs 0.037 mg/L)^8^, yeast (8 h EC_50_ in *Saccharomyces cerevisiae*, 121 vs 134 mg/L)^2^, protozoa (24 h EC_50_ in *Thamnocephalus platyurus*, 0.18 vs 0.24 mg/L, and *Tetraphymena thermophile*, 6.8 vs 7.4 mg/L)^1^,^3^, crustaceans (48 h LC_50_ in *Daphnia magna*, 3.2 vs 8.8 mg/L)^3^, and fish (96 h LC_50_ in zebrafish, 4.92 vs 3.31 mg/L)^7^. These phenomena revealed that ZnO NPs lacked nano-specific toxicity, which conflicted with our general understanding that nanoparticles represented more serious hazardous threats to living organisms than bulk particles. Based on these results, whether ZnO NPs represent a similar hazard to the environment as bulk ZnO and whether the toxicological assessment pattern applied for bulk ZnO is also suitable for nano ZnO are unknown.

A previous study reported the nanotoxicities of metal and/or metal oxide NPs derived from either NPs themselves, released metal ions, or both. For ZnO NPs, the released Zn^2+^ was proposed to be predominantly responsible for the observed toxicity based on the comparison of the toxicity of ZnO particles with zinc salts. However, numerous studies demonstrated that the ZnO particle solubility percentages were 15% lower in deionized or distilled water^9^,^10^,^11^,^12^, indicating that the dissolved Zn^2+^ concentration from the ZnO particles was dramatically lower than the dissolved Zn^2+^ from the applied zinc salts. Indeed, recent evidence showed that dissolved Zn^2+^ from ZnO NPs only partially contributed to acute toxicity in the A549 cell line^13^, *Danio rerio*^9^, *E. coli*^10^ and *D. magna*^14^. Indeed, Zn^2+^ was responsible for approximately 10% of the overall toxicity in the A549 cell line and 31% in *D. magna*^13^,^14^. Thus, the conclusion that the mechanism underlying NPs toxicity was primarily dependent on the ions from released the NPs and metal salt was an oversimplification. Importantly, the concentration of dissolved metal ions should be measured and reported in the toxicological study and environmental risk assessment.

The yeast *Saccharomyces cerevisiae* is a unicellular eukaryotic model organism, and its mutants are widely employed to study the toxicity of nanomaterials^2^,^15^,^16^. Mutant strains with a deleted gene or genes are used to induce deficiencies in the defense mechanisms, and these mutants show stronger sensitivity and specificity than the wild type strain (BY1437)^15^,^16^. As previously described^15^,^16^, the *yap1* mutant (*yap1*Δ) has a single deletion in the *yap1* gene, which regulates the enzymatic response to oxidative stress; this mutant is sensitive to NPs-related oxidative toxicity. The quadruple gene deletion mutant (4Δ; *cwp1*Δ *cwp2*Δ *snq2*Δ *pdr5*Δ) controls cell membrane permeability and is sensitive to metal ions or mechanical damage-induced toxicity. The quintuple gene deletion mutant (5Δ; *cwp1*Δ *cwp2*Δ *snq2*Δ *pdr5*Δ *yap1*Δ) governs both the effect of oxidative defense and cell membrane permeability and is sensitive to oxidative and/or mechanical damage toxicity. Thus, based on their sensitivities and specificities, these three yeast mutants (*yap1* mutant (*yap1*Δ), quadruple mutant (4Δ) and quintuple mutant (5Δ)) were employed to determine the toxicity and potential nano-specific toxicity of ZnO NPs.

We investigated the cytotoxicity of ZnO NPs to the yeast *S. cerevisiae* and its three mutants, determining the mechanism underlying the toxicity of ZnO NPs in the yeast. The additional purposes of the present study were to investigate the potential nano-specific toxicity of ZnO NPs and to serve as an environmental risk assessment. The underestimated nano-specific toxicity of ZnO NPs was confirmed in the mutant strains, indicating an enhanced toxic risk in humans and possibly the environment. Moreover, toxicity enhancements (*T*_e_^(particle)^ and *T*_e_^(ion)^) were proposed to assess the NPs nano-specific toxicity and to determine whether the toxicity was dependent on the dissolved ions.

## Results and Discussion

### Characterization of ZnO Particles

The physiochemical properties and TEM images of the nano and bulk ZnO particles are displayed in Table S1 and Fig. S1, respectively. Most of the ZnO NPs were 20 nm in size as described in the manufacturer’s information. However, most of the ZnO bulk particles belonged to the microsize scale (>200 nm).

### ZnO (nano and bulk) Particles and Zinc Salt Toxicity towards *Saccharomyces cerevisiae*

The toxic effects of ZnO particles and zinc salt in a set of *S. cerevisiae* strains are expressed as EC_50_ values in Table 1 and Fig. S2. As expected, the ZnO NPs showed hazardous effects that were analogous to the bulk ZnO in wild type yeast (8.83 vs 11.66 mg/L; *T*_*d*_ = 2.83 mg/L and *T*_*e*_ = 1.32). The same tendency in yeast was reported previously (*T*_*e*_ = 1.11)^2^, although the toxic values were almost 100-fold higher in this study. The difference could have two possible explanations: the first is the use of different endpoints and exposure durations (growth inhibition vs cell viability and 8 vs 6 h, respectively); the second reason is that different testing conditions were used (growth medium vs deionized water). Importantly, fruitful proteins in the growth medium but not the deionized water could coat the surfaces of the ZnO NPs and hinder their cytotoxicity^17^. These results revealed that the nano-specific toxicity of ZnO NPs was undetectable. As discussed above, this phenomenon was demonstrated in several studies regardless of the test organisms, toxic endpoints and exposure durations (see Table S2). The predominant difference between nano and bulk ZnO is the particle size. According to the investigation of the size-dependent toxicity of ZnO NPs, the ZnO NPs toxicity was not governed by particle size or its related factors. The toxicity was significantly more serious compared to other nano or bulk ZnO particles only when the NPs size was less than 20 nm^12^,^18^. The possibility of aggregation in the present investigation suggested that the ZnO NPs toxicity was not dependent on the particle size, which was reported to strongly affect Zn^2+^ release^19^. Thus, the dissolved Zn^2+^ could not be strongly correlated with the toxicity (as detailed below). Notably, in the present study some of the ZnO bulk particle sizes still belonged to the nanoscale (<100 nm, see Fig. S1).

Herein, the mutant strains exhibited varied toxicity patterns in BY4741. Increased differences in toxicity were observed when the nano ZnO was compared with bulk ZnO (*yap1*Δ: *T*_*d*_ = 43.15 mg/L; 4Δ: *T*_*d*_ = 20.70 mg/L and 5Δ: *T*_*d*_ = 13.75 mg/L). We hypothesized that these increased toxicity differences were ZnO NPs nano-specific. Previously, single gene deletion yeast mutants were used to examine the nanotoxicity of CuO NPs. The *yap1*Δ mutant showed higher tolerance to CuO NPs toxicity^15^,^16^. This result was consistent with our result that nano and bulk ZnO yielded lower toxicity in *yap1*Δ, which was reflected by the higher EC_50_ compared to the wild type. Moreover, *yap1*Δ showed similar tolerance to other NPs, such as Ag NPs and Ag_2_O NPs (data not shown). Theoretically, *yap1*Δ should be sensitive to ROS-mediated toxicants. Indeed, intercellular ROS investigations confirmed that the *yap1*Δ strain was sensitive to ZnO NPs (see below), and CuO NPs induced more ROS than the wild type strain^16^. The comparative toxicities of these NPs in *yap1*Δ and the wild type strain could possibly be explained by their toxic mechanisms, which slightly accounted for ROS generation. However, the rigid cell wall of the yeast can prevent the nano or bulk particles from entering the intercellular space and stop the triggering of the deficient defense system that is hypersensitive to oxides such as hydrogen peroxide. Thus, in this instance, the observed toxicity is only governed by mechanical damage, such as that described here. Alternatively, the reported compensatory mechanism in the cell may mask the deficiency of a single gene’s desired phenotype^20^,^21^. Correspondingly, multiple gene deletion mutants can be more sensitive and adequate for cytotoxicity estimations. As expected, the quadruple (4Δ) and quintuple (5Δ) mutants were more sensitive than the wild type and *yap1*Δ strains to ZnO NPs with lower EC_50_ values (6.02 and 4.34 mg/L, respectively). A similar phenomenon was observed for CuO NPs^16^, suggesting the sensitivity and specificity of these mutant strains to nanotoxicity. Considering the deficiencies in the 4Δ and 5Δ gene mutants^20^,^21^, the observed serious toxic effects could be assumed to correlate with ROS and/or cell membrane permeability. Furthermore, although they presented decreased toxicity differences (26.70 and 13.75 mg/L, respectively), their nano-specific toxicities were still detectable (log *T*_*e*_^(particle)^ ≈ 0.65). Interestingly, lower toxic effects were observed for the 4Δ and 5Δ mutants during contact with bulk ZnO compared with the wild type yeast. However, further investigations are needed to understand the phenomenon.

### Mechanisms underlying the nano-specific toxicity of ZnO NPs

Three major mechanisms were previously proposed to be involved in the toxic actions of metals and metal oxide NPs^5^,^6^,^22^: (I) Released metal ions from particles (e.g., Zn^2+^ from ZnO NPs); (II) ROS generated from interactions between NPs and biological targets (e.g., **·**OH or O_2_^•−^); and (III) mechanical damage due to the direct interactions of NPs with biological targets (e.g., broken cell membranes). Herein, the three toxic mechanisms were examined and detailed below individually to elucidate the mechanism by which ZnO NPs induce nano-specific toxicity in organisms.

### Dissolved Zn^2+^

Recently, many scientists reported that the mechanism underlying the toxicity of ZnO NPs was dependent on the release of Zn^2+^. Indeed, many investigations found that ZnO NPs displayed toxicity in organisms comparable to those targeted with bulk ZnO and zinc salts (ZnCl_2_ or ZnSO_4_·7 H_2_O). Nevertheless, as noted previously the Zn^2+^ concentrations released from ZnO NPs were too low to explain the toxic injury^9^,^23^, indicating that additional mechanisms (i.e., solid ZnO NPs) played a role in the toxic effect.

**Figure 1:**
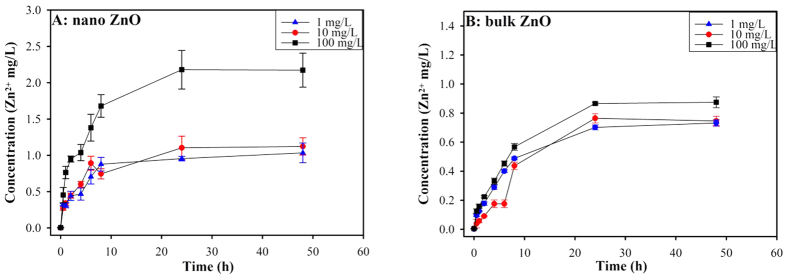
Dissolution curve of Zn^2+^ from different concentrations of nano ZnO (A) and bulk ZnO (B) in deionized water as a function of time. The data were presented as the mean ± SD from three replications (n = 3).

**Figure 2:**
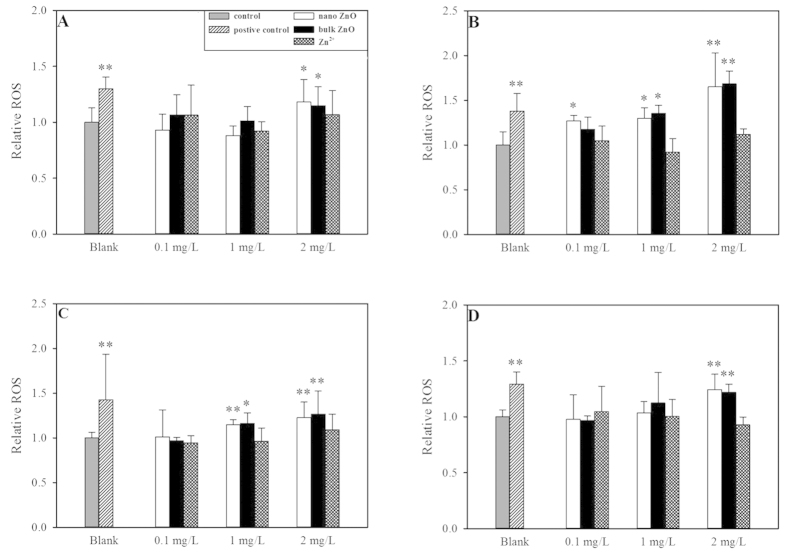
Relative intercellular ROS generated by three selected concentrations of ZnO NPs, bulk ZnO and Zn^2+^ (ZnSO_4_•7H_2_O). H_2_O_2_ was used as the positive control. (**A**) BY4741; (**B**) yap1Δ; (**C**) 4Δ; (**D**) 5Δ. Asterisks (*and **) denote significant and extremely significant differences compared to the control group (p < 0.05 and p < 0.01), respectively. The data were presented as the mean ± SD from five replications (n = 5).

As shown in Fig. 1, the Zn^2+^ release curves for nano and bulk ZnO were very close at the 1 and 10 mg/L concentrations, although more Zn^2+^ was released from nano ZnO than bulk ZnO at the highest concentration (100 mg/L). At the 1 mg/L concentration, nano ZnO dissolved rapidly and completely. This finding was confirmed by the Noyes-Whitney equation^24^. In contrast, less than 10% of the nano and bulk ZnO were dissolved at the primary concentrations of 10 and 100 mg/L, which was consistent with previous reports^9^,^10^. Both nano and bulk ZnO illustrated stronger toxic effects in the set of yeasts than the zinc salt (ZnSO_4_·7 H_2_O) (see Fig. S2 or Table 1). Thus, the Zn^2+^ released from either the ZnO NPs or bulk ZnO was not sufficient to elicit serious toxicity. Even when a high concentration of Zn^2+^ was employed (101,290.00 mg/L), a less than 10% decrease in cell viability was detected in the quadruple mutant (4Δ). The more than two log unit higher toxicity of the nano and bulk ZnO strongly indicated that the mechanism underlying the toxicity in yeast was essentially dependent on the particles themselves and not on the released zinc ions. However, we evaluated the extent to which the toxic effect was attributable to the dissolved zinc ions using previously described methods^14^. As shown in Fig. S3, the calculated concentrations of nano and bulk ZnO led to an almost 50% decrease in cell viability, with EC_50_ ranges from 44.16 to 57.35%. This result indicated that the cell viability testing was valid. Moreover, Zn^2+^ released from either nano ZnO or bulk ZnO yielded less than 9% cell death (range 3.16 to 8.25%, see Fig. S3). Correspondingly, the contributions of Zn^2+^ to the overall toxic effect accounted for less than 8% (range 2.93 to 7.44%; see Table 2). Zn^2+^ sometimes did not induce toxic effects in the yeast or mutants, resulting in the larger error bars in Fig. S3. Moreover, the investigation of intercellular ROS demonstrated that dissolved Zn^2+^ did not induce significant oxidative stress in the yeast (p > 0.05) compared to the ZnO particles (see Fig. 2). Because the solubilities of metals and metal oxides vary and most metal ions induce diverse toxic effects in organisms, the concentrations of metal ions released from NPs should be routinely measured and provided in the ecotoxicological and environmental assessments.

Additionally, the intercellular Zn^2+^ could be another toxic source. Theoretically, 4Δ mutant with cell membrane permeability defect should be more sensitive to free ion-related toxic effect than BY1437, because the released Zn^2+^ could be inside of 4Δ mutant freedom. Therefore, the concentration of intercellular Zn^2+^ should be higher in 4Δ mutant cell than in BY1437 cell. Once the intercellular Zn^2+^ can induce serious toxic effect, the applied lower concentration of zinc salt should be inducing serious toxic effect in 4Δ mutant. However, when a high concentration of Zn^2+^ was employed (101,290.00 mg/L), a less than 10% decrease in cell viability was detected in the 4Δ. Furthermore, the applied comparable free Zn^2+^ (ZnSO_4_·7H_2_O) did not induce severe intercellular oxidative stress (p > 0.05, see Fig. 2) in the yeast strain. Comparably, the ZnO NPs caused significant intercellular oxidative stress (p < 0.05 or 0.01). The combination of these results revealed that the toxic effect of nano or bulk ZnO was not mainly dependent on the released Zn^2+^ but on particle-related toxicity (i.e., oxidative stress and/or mechanical damage). Importantly, the particles contributed to the comparable toxic effect in the set of yeasts (almost 95%, see Table 2); hence, the dissolved Zn^2+^ was not the essential distinction for understanding why the ZnO NPs did not induce higher toxicity than bulk ZnO.

### Intercellular ROS

In addition to the dissolved metal ions, ROS generation was proposed to be a general paradigm for the toxic action of metal and metal oxide NPs^5^,^10^,^25^. The induced intercellular ROS could lead to dysfunctions in mitochondrial oxidative phosphorylation and ultimately cause cell death. Therefore, intercellular ROS generation was explored to evaluate whether oxidative stress was the major toxic mechanism of ZnO particles and the inherent factor in nano-specific toxicity elicitation. Our previous investigation demonstrated that the multiple gene-deleted mutants were sensitive to NPs-generated oxidative stress^16^. As shown in Fig. 2, the selected highest concentration (2 mg/L) of ZnO particles generated significantly (compared to wild type) and extremely significantly (compared to the other mutants) higher ROS than the control group (p < 0.05 and p < 0.01). Comparably, 0.1 mg/L of ZnO particles did not induce serious oxidative stress in the yeast, although significant oxidative stress was detected in the *yap1*Δ mutant following exposure to 0.1 mg/L of ZnO NPs (p < 0.05, see Fig. 2B). Oxidative stress in these yeast strains was aggravated by the increasing concentrations of ZnO particles, suggesting that oxidative damage at least partially contributed to the toxic effect. Similarly, ZnO NPs exerted antibacterial activity against *S. cerevisiae* via the induction of oxidative stress^12^.

Interestingly, nano and bulk ZnO showed similar patterns of oxidative stress in the yeast mutants, with either no extraordinary oxidative stress at the 0.1 mg/L concentration (expect for *yap1*Δ) or (extremely) significantly high oxidative stress at the 2 mg/L concentration. The lack of significant differences in the effects of the ZnO particles on the yeast strains (p > 0.05) suggested that oxidative stress was not an essential characteristic to differentiate ZnO NPs nano-specific toxicity.

Notably, extracellular ROS generated in response to the interaction of the particles with cellular membranes (walls) also contributed to the antibacterial activity of ZnO NPs^26^,^27^. As reported earlier, particle-dependent ROS formation perturbed electronic transfer processes in the cell, such as those in the mitochondrial inner membrane^23^. Extracellularly generated ROS can also lead to cell membrane damage, cell lysis and ultimately cell death^28^,^29^. Theoretically, the primary location for extracellular ROS generation is the yeast cell wall. This conclusion can be confirmed by the data of zeta potentials of the particles and yeast strains (see below). Hence, the generated ROS from solid ZnO on the surface of the yeast walls could be an additional toxic source in this study. The extracellular ROS could possibly serve as helper to destroy the cell walls or membranes, and then enter into the internal yeast cell. Indeed, Fig. S4 suggested that the yeast cell wall and membranes were partly destroyed. Although without of ZnO NPs adhering to the cell wall or membrane was found through TEM, extracellular ROS might be generated at the extracellular wall to some extent. Perhaps, the ZnO NPs could be adhered on the surface of the yeast cell walls, but the microenvironment could promote dissolution of ZnO NPs on the cell wall, resulting shift of particle-related toxic effect to ion-related toxic effect. Therefore, the ROS or other relatedly biological mechanisms could attribute to the cytotoxicity, although the possibly toxic effects were not quantitatively measured in this study.

#### Mechanical damage

**Figure 3:**
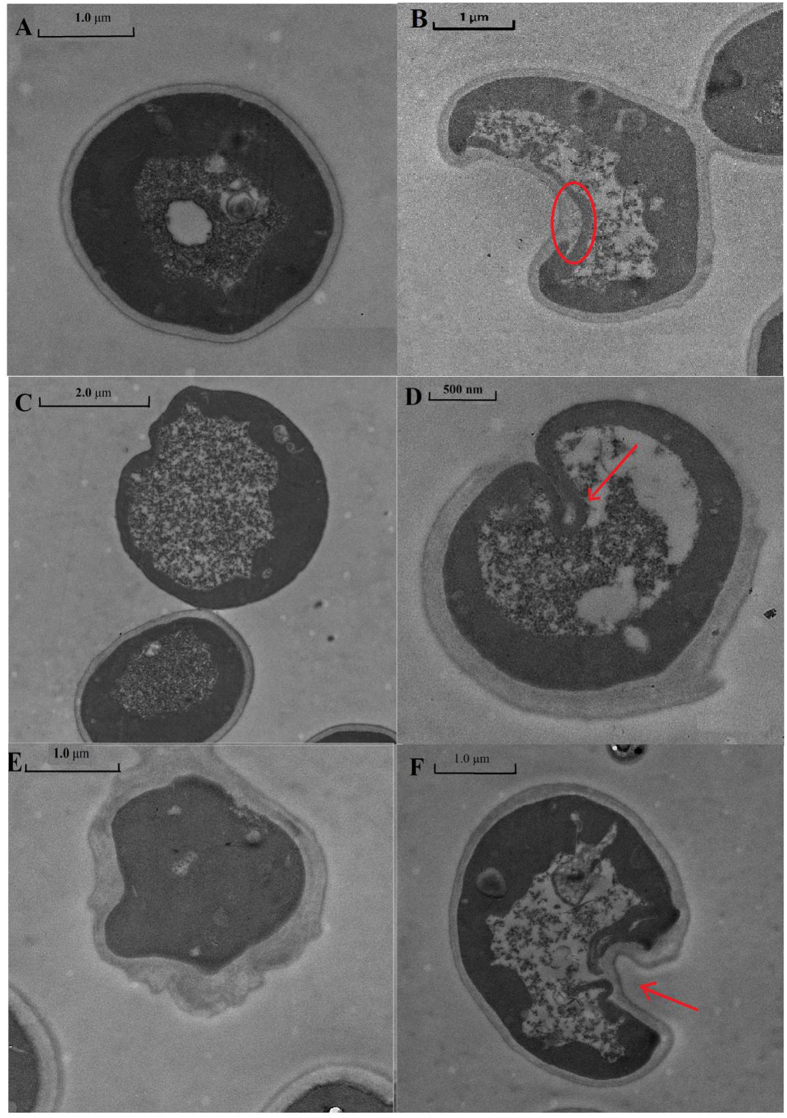
The morphology of yeast strain cells exposed to nano ZnO (10 mg/L). (**A**) normal cell, (**B**) cell cytoplasm leakage, (**C**) crushed cell wall, (**D**) partially crushed cell wall, (**E**) irregularly shaped cell, (**F**) deformed cell with sunken area.

The TEM images indicated that the yeast morphologies following ZnO NPs exposure were dramatically different compared with normal yeast cells (see Fig. 3). The cell walls of some of the yeast were broken or partially broken (Fig. 3C,D). The phenomenon was also observed in *E. coli*^29^. In the other yeast, although the cell walls were not crushed completely, the ZnO NPs-treated cell walls were deformed with some sunken areas (Fig. 3F) or deficiencies (Fig. 3E). Figure 3E indicated that the yeast cell walls were disrupted and that the morphology of the normal cell changed from globular to an irregular shape.

Regardless, the rigid yeast cell wall could protect the yeast cells from ZnO NPs-induced mechanical damage. Moreover, as shown in Fig. S4, the yeast cell wall prevented the passage of the Annexin V-FITC dye (Q4 region) but not PI (Q1 region), suggesting that most of the rigid cell walls were not crushed. This cell wall protection was also reported for several other organisms, such as *S. typhimurium, K. pneumonia, and S. aureus*^29^, in which breakage of the cell wall or membrane was not detected. Correspondingly, these sunken areas suggested that endocytosis could be a potential route for the entrance of ZnO NPs into the cell. However, the cell membrane was fragmented under the sunken areas as indicated with the red arrow in Fig. 3D,F. The disorganization or permeability of the yeast membrane during nano and/or bulk ZnO exposure can lead to the loss of cell cytoplasm and ultimately to cell death. As shown in Fig. 3B, the cell cytoplasm leaked and passed through the cell membrane, although the rigid cell wall prevented the cytoplasm from leaking out. Furthermore, without of intercellular ZnO NPs into cell or adhesion of ZnO particles on cell wall or membrane were detected by TEM in this study. These founding suggested that the ZnO particles could not be taken into by the yeast strains. Comparably, ZnO NPs exposure led to *Escherichia coli* cell membrane fragmentation and deformation^10^,^30^. Without the protection of the tough cell wall, ZnO NPs were detected in the inner bacterial cell^29^,^30^,^31^. Furthermore, ZnO NPs arrived in certain fish organs through waterborne routes or ingestion and subsequently caused tissue damage^7^,^32^. For example, ZnO NPs induced shrinkage and loss of the cell cytoplasm in zebrafish gills^7^, hyperplasia of the gill filament epithelium and edema of the carp gill lamellae^33^. ZnO NPs were also observed to alter the structural and functional integrity of cell junctions in the *Xenopus laevis* intestine^32^. Despite the dissolved Zn^2+^ was not shown to induce serious cytotoxicity here, the “Trojan horse effect” derived from the internalized NPs was seriously considered^34^,^35^. Although we examined the yeast cells very carefully by TEM, no intercellular ZnO particles were found. This result suggested that the ZnO particles can not be taken by the yeast strains. Interestingly, our resent study found similar phenomenon for CuO NPs that no CuO NPs was inside yeast cells under same cultivation^16^. Even if a very little amount of ZnO particles could enter the yeast cell, they should rapidly dissolve in the acidic environment of the lysosome (pH 5). Based on the combination of qualitative inspection by TEM and the quantitative data presented in Fig. S4 (Q4 region), the rigid cell wall of the yeast was barely broken and the ZnO particles scarcely entered the intercellular area of the yeast cells. Therefore, the “Trojan horse effect” was not validated to yeast strain in this study. Theoretically, determining the uptake routes of ZnO NPs into organisms with or without of cell walls are very important for the toxicity inducement and risk assessment.

**Figure 4:**
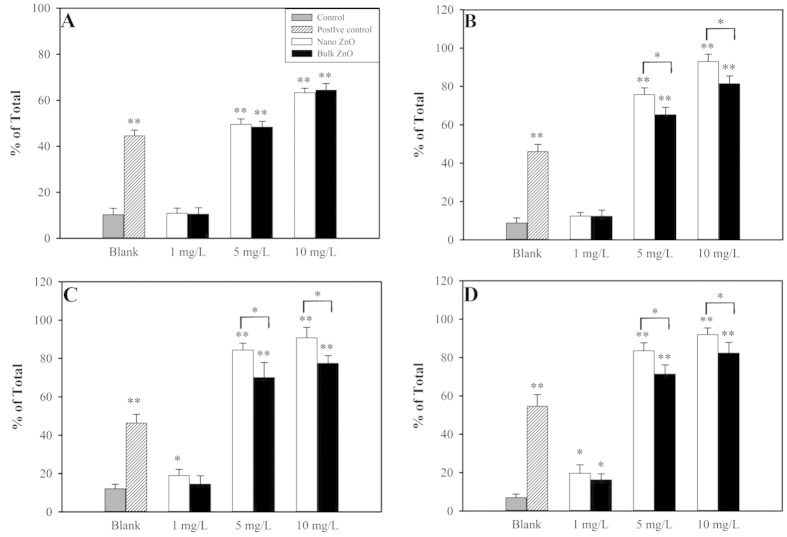
Cell membrane permeability caused by the 1, 5 and 10 mg/L concentrations of nano ZnO and bulk ZnO. H_2_O_2_ served as the positive control. (**A**) BY4741; (**B**) *yap1*Δ; (**C**) 4Δ; (**D**) 5Δ. Asterisks (* and **) denote significant and extremely significant differences compared to the control group (p < 0.05 and p < 0.01), respectively. The data were presented as the mean ± SD from six replications (n = 6).

Undoubtedly, the mechanical damages caused by particles binding to the cell surface are majorly governed by electrostatic forces, and hence the zeta potentials of the particles and yeast strains were measured (see Table S1). All yeast strains showed the expected negative charge, and all of their negative charges were slightly decreased at the 6 h time point. Interestingly, the ZnO NPs possessed a positive charge that was absolutely different from the negative charge of the bulk ZnO. The yeast strains preferred to bind to the ZnO NPs but not to the bulk ZnO. Theoretically, more serious cell wall and/or membrane damage would emerge under ZnO NPs exposure compared to bulk ZnO. Indeed, this finding was confirmed by the PI dye experiment shown in Fig. 4, in which ZnO NPs at concentrations of 5 and 10 mg/L induced stronger cell permeability in *yap1*Δ, 4Δ and 5Δ compared to bulk ZnO (p < 0.5). Interestingly, no significant difference in cell permeability was detected between the nano and bulk ZnO in the wild type strain (p > 0.5, see Fig. 4A). One possible explanation was that the mechanical disturbance under the shaking condition (200 rpm) overcame the electrostatic forces and contacts that existed between the bulk ZnO and the cell wall.

Massive ROS species generation could also lead to cell membrane damage and ultimately cell death^28^,^36^. In the present investigation, intercellular ROS generation could not possibly lead to cell membrane damage because the relative ROS concentrations generated during exposure to ZnO NPs and bulk ZnO were comparable (p > 0.05) in each yeast strain. In contrast, the mechanical damage showed different patterns between the wild type and mutants that could primarily account for their various physiologies. The deletion of a gene or genes affected the cell defense mechanism, which was very sensitive and specific for nanotoxicity. In the wild type yeast, the sensitivity or specificity could be masked by an influencing factor, such as the shaking described here.

In summary, the free Zn^2+^ released from ZnO (nano and bulk) particles were just slightly contributed to the detected cytotoxicity. Oxidative damage, possibly from both extra- and inter-cellular ROS, partially contributed to the toxic effect of the ZnO particles. Additionally, mechanical damage was another mechanism underlying the toxicity of ZnO NPs and most likely accounted for the nano-specific toxicity of ZnO NPs in the mutants.

### Toxicity Differences and Toxicity Enhancement of ZnO NPs in the Wild Type and Mutants

As summarized in Table 1, the ZnO particles displayed similar cytotoxicity in the wild type yeast (EC_50_: 8.83 (nano) vs 11.66 (bulk) mg/L; *T*_*d*_ = 2.83 mg/L). However, different cytotoxicity patterns between bulk ZnO and nano ZnO were detected in the mutants (*T*_*d*_ = 43.15 mg/L for *yap1*Δ, *T*_*d*_ = 26.70 mg/L for 4Δ and *T*_*d*_ = 13.75 mg/L for 5Δ). Therefore, the nano-specific toxicities of ZnO NPs in the yeast were not exhibited towards the wild type but were exhibited towards the three mutants. There was a decreasing tendency for nano-specific toxicity in BY1437 < 5Δ < 4Δ < *yap1*Δ. However, the tendency conflicted with the toxicity results, where 5Δ presented the highest sensitivity to ZnO NPs, followed by 4Δ, BY 1437 and *yap1*Δ. Indeed, we previously observed that *yap1*Δ was less sensitive to NPs toxicity, and the ZnO NPs led to the lowest cytotoxicity for *yap1*Δ herein (see Table 1). The strongest cytotoxicity of the ZnO NPs was detected for the 5Δ strain, which exhibited almost two-fold higher toxicity than the wild type. These observed toxicity differences were due to the strains’ distinct physiologies and related toxic mechanisms (as discussed above). Compared to the wild type, the three mutants lacked sensitivity to bulk ZnO, which had a higher EC_50_ (11.656 vs 55.82 vs 26.72 vs 18.09 mg/L).

In parallel with the toxicity differences, toxicity enhancement (*T*_*e*_) is another important parameter to evaluate the nano-specific toxicity of NPs. From the organic chemistry toxicity perspective, toxicity enhancement (*T*_*e*_) had been successfully utilized to discriminate and evaluate the excess toxicity of organic chemicals^37^. *T*_*e*_ = 10 was identified as a threshold to separate excess toxicity (log *T*_*e*_ ≥ 1) from baseline toxicity (log *T*_*e*_ < 1). Theoretically, nanoparticles should exert seriously harmful damage compared to their bulk counterparts with identical chemistry because the toxicity is nano-specific. Thus, the nano-specificity of the NPs toxicity could be expressed as the toxicity enhancement. In the present investigation, a hypothesis was proposed that bulk particles only exerted baseline toxicity but NPs possibly exhibited excess toxicity due to their unique physicochemical properties. Therefore, it is likely that the nano-specific toxicity of NPs in the target organisms can be expressed as excess toxicity compared with the bulk particles.

As shown in Table 1, the log *T*_*e*_^(particle)^ for the wild type yeast was 0.12, indicating that no excess toxicity was found. Thus, the ZnO NPs exhibited no nano-specific toxicity for the wild type yeast. Not only was this phenomenon previously found in wild type yeast but similar results were also reported for other organisms. As shown in Table S2, the log *T*_*e*_^(particle)^ of the ZnO NPs was less than 0.5, suggesting that the ZnO NPs induced a level of toxicity similar to bulk ZnO but did not induce nano-specific toxicity. In contrast, CuO NPs are an excellent example of NPs that induce nano-specific toxicity in target organisms (see Table S2). Regardless of the target organisms, toxic endpoints or exposure duration, log *T*_*e*_^(particle)^ > 1 of the CuO NPs indicated excessive toxicity compared to bulk CuO. Interestingly, all three mutant strains possessed dramatically similar log *T*_*e*_^(particle)^ values (0.64 vs 0.65 vs 0.62). Similar to the *T*_*d*_, *T*_*e*_^(particle)^ showed that the ZnO NPs exerted at least partial nano-specific toxicity for the mutant strains, although the *T*_*e*_^(particle)^ was < 10. Importantly, the ZnO NPs yielded almost identical nano-specific toxicities in the mutant strains, revealing that the three mutants possessed almost identical sensitivity and specificity for the ZnO NPs nano-specific toxicity. *T*_*e*_^(particle)^ can exactly reflect the excess NPs toxicity in terms of the toxicity difference, although the latter parameter considers the specificity of the physiology of diverse strains.

Different from *T*_*e*_^(particle)^, *T*_*e*_^(ion)^ exhibited a varied pattern of excess toxicity in the wild type yeast and mutants. The log *T*_*e*_^(ion)^ covered almost 3 log units (from 1.35 (BY) to 4.23 (4Δ)), suggesting that ZnO NPs induced extremely excessive toxicity compared to Zn^2+^. Moreover, the log *T*_*e*_^(ion)^ was >1 herein, which strongly indicated that the mechanism underlying the toxicity of the ZnO NPs in the yeast was not dependent on dissolved Zn^2+^. As shown in Table S2, different results suggested that the ZnO NPs exhibited toxicity that was comparable with Zn^2+^. Indeed, the log *T*_*e*_^(ion)^ equaled zero for *T. thermophile* and *P. subcapitata*, indicating that the toxicities of the ZnO NPs and zinc salt were identical. The log *T*_*e*_^(ion)^ in *V. fischeri* was negative 0.24, suggesting the Zn^2+^ showed slightly higher toxicity than the ZnO NPs. As mentioned above, the possible dissolved ratio of Zn^2+^ in the NP suspensions was not sufficient to induce toxicity to a level comparable with the zinc salt. Thus, it was an oversimplification to assert that the mechanism underlying the ZnO NP toxicity was primarily dependent on dissolved Zn^2+^ based on the similar toxic effect observed for the ZnO NPs and zinc salt. Enrique Navarro *et al*. corrected this shortcoming by examining the functions of metal ion concentrations^38^. Importantly, log *T*_*e*_^(ion)^ = −1.24 (18-fold higher toxicity for AgNO_3_ than for Ag NPs) confirmed the finding that the released Ag^+^ was a major toxicity resource, although Ag^+^ could not fully explain the toxicity in *Chlamydomonas reinhardtii*. Moreover, based on the inhibition in *E. coli* following exposure^39^, a log *T*_*e*_^(ion)^ < −1 for Ag NPs was proposed that supported the reported result that the toxicity was directly correlated with the released metal ions. Furthermore, comparable log *T*_*e*_^(ion)^ values (−0.89 vs −0.91, very close to −1.00) were calculated for two differently coated Ag NPs in *Eisenia fetida*, thereby confirming that the proposed mechanism of toxicity was dependent on the released Ag^+ ^40.

The CuO NPs exhibited varied toxicity patterns compared with the ZnO NPs. With the exception of *S. cerevisiae*, the log *T*_*e*_^(ion)^ values of the CuO NPs were larger than negative 1.30, revealing that Cu^2+^ showed more than 10-fold higher toxicity than the CuO NPs. The different log *T*_*e*_^(ion)^ values of the ZnO and CuO NPs strongly indicated that the mechanisms underlying the ZnO NPs toxicity were not associated with the released Zn^2+^, whereas the mechanism underlying the CuO NPs toxicity was associated with the released Cu^2+^. Notably, differences in the log *T*_*e*_^(ion)^ values between the ZnO and CuO NPs could also occur because Zn^2+^ showed less toxicity compared with Cu^2+^. The Zn^2+^ and Cu^2+^ concentrations that induced growth toxicity ranged from 200 to 300 and 15 to 20 μg/g dry weight, respectively^41^. Moreover, Cu^2+^ formed more stable complexes with proteins and/or peptides than Zn^2+ ^42, suggesting that the Cu^2+^ -mediated toxicity was more irreversible and serious. In *S. cerevisiae*, the log *T*_*e*_^(ion)^ of the CuO NPs was an outlier at 0.12, indicating that the toxicity of the CuO NPs almost equaled the toxicity of Cu^2+^. This result was most likely caused by the testing circumstances: growth medium (log *T*_*e*_^(ion)^ = –0.10, growth inhibition) vs deionized water (log *T*_*e*_^(ion)^ = −0.77, cell viability)^15^. Based on our unpublished cell viability data, the log *T*_*e*_^(ion)^ of CuO NPs in deionized water is −0.97, suggesting that the effect of Cu^2+^ on cell viability is almost 10-fold higher than the effect of CuO NPs. As mentioned above, proteins in the growth medium could strongly capture Cu^2+^ and hence decrease the toxicity of Cu^2+^ 15.

These findings suggested that the mechanism underlying the ZnO NPs toxicity was not mainly related to the released Zn^2+^. The present results revealed that log *T*_*e*_^(particle)^ could be a useful parameter to evaluate whether certain NPs induce nano-specific toxicity in target organisms, whereas log *T*_*e*_^(ion)^ could be employed to understand whether the mechanism of the toxicity of the metal or metal oxide NPs correlated with the corresponding dissolved metal ions.

## Conclusions

In summary, ZnO NPs displayed nano-specific toxicity in the yeast mutants, although no nano-specific toxicity was observed in the wild type yeast. Oxidative damage partially contributed to the adverse effects in the yeast strains but did not contribute to the nano-specific toxicity of the ZnO NPs in the mutants. Mechanical damage also attributed to the adverse effects of the ZnO NPs in the yeast strains and was the essential mechanism underlying the nano-specific toxicity in the mutants. The nano-specific toxicity of the ZnO NPs’ could potentially lead to serious environmental hazards and threaten human health; thus, the underestimated nano-specific toxicity should be carefully considered in the risk assessment. In particular, log *T*_*e*_^(particle)^ is a useful parameter to estimate the NPs’ nano-specific toxicity in target organism, whereas log *T*_*e*_^(ion)^ is an efficient parameter to determine whether the NPs’ toxicity mechanism is primarily correlated with its released ions.

## Methods

### Test organisms and chemicals

The basic information for the yeast *S. cerevisiae* strains is summarized in Table S1. The cultivation conditions and procedures for the *S. cerevisiae* strains were detailed previously^16^. The viability of the yeast strains and the effect of the pH value during the growth phase were explored previously^16^ and were shown in Figs S5 and S6. The cell viabilities of these yeast strains under non-stress conditions at different time points were measured and displayed in Fig. S7. The combination of the above information and the cell viabilities of selected strains were not significantly different under non-stress conditions, and hence the detected cytotoxicity was due to the employed chemicals.

ZnO NPs (20 nm) were purchased from Beijing Nachen S &T Ltd..; the purity was 99.9%. Bulk ZnO and ZnSO_4_·7H_2_O, with over 99.9% purity were purchased from Tianjin Kemiou Chemical Reagent Co. Ltd., China. Considering the great purity of these materials, the toxic effects detected in this investigation were derived from the materials.

The shape and size of the ZnO particles in suspension were visualized by transmission electron microscopy (TEM, JEM-100CXII, JEOL, Ltd., Japan). The released Zn^2+^ from three concentrations (1, 10 and 100 mg/L) of ZnO particles in the absence of light at room temperature was detected by Atomic Absorption Spectroscopy. The zeta potentials and hydrodynamic diameters of the ZnO particles and the zeta potentials of the yeast strains were characterized at the 1 and 6 h time points using a Nano-Zetasizer (1000HS, Malvern Instrument Ltd., UK).

### Cytotoxicity test

Both nano and bulk particles were prepared at high concentrations in deionized water and then stored in the dark after ultrasonication for 30 min. Similarly, ZnSO_4_·7 H_2_O was employed for Zn^2+^ stock solution preparation without ultrasonication.

The cytotoxicity test for yeast cell viability was performed as follows. Yeast in the logarithmic growth phase after overnight cultivation were harvested and washed three times with sterile deionized water. The yeast pellets were resuspended and diluted using sterile deionized water until the cell density (optical density, OD_600_) was equal to 1.050 (±0.030). Nominal concentrations of the test compounds were obtained through the 10-fold dilution method. Under sterile conditions, at least six different concentrations (with three replications) and a blank control were evaluated. After six hours of exposure (200 rpm, 30 °C), 100 μL of the test suspension for each concentration was diluted and spread onto YPD solid plates. The colonies were counted after two days of growth and compared with the control.

The EC_50_ values were calculated using dose-response cytotoxicity curves generated from a confirmed four-parameter sigmoid function equation (1) 37:


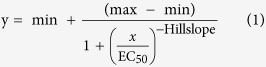


The calculated EC_50_ values of the nano and bulk ZnO served to determine the relative contributions of the nano and bulk particles and their released zinc ions to the cytotoxicity of the yeast and mutants. Part of a suspension with a nominal EC_50_ was utilized to examine the overall inhibition. The residual of the suspension was centrifuged (10,000 x g, 10 min) and then the supernatant was passed through a 0.22 μm filter to obtain the released Zn^2+^. The released Zn^2+^ was applied to explore the ions’ contribution to the cytotoxicity. The details of the toxicity exposure were the same as described above. The relative contributions to the cytotoxicity from the particles or ions were obtained through equation 2 based on the hypothesis that ZnO particles and Zn^2+^ possessed different modes of action as reported previously^14^.


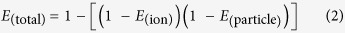


in which *E*_(total)_ indicates the total detected toxic effect derived from the suspensions, *E*_(ion)_ indicates the detected toxic effect from the dissolved Zn^2+^, and *E*_(particle)_ denotes the nano or bulk ZnO particle-induced toxic effect.

### Intracellular ROS

Intracellular ROS generation resulting from contact with the ZnO NPs, bulk ZnO and Zn^2+^ was detected using the fluorescent probe 2′,7′-dichlorofluorescin diacetate (H_2_DCFDA). After 6 hours of toxic exposure, the yeast cells were harvested and washed three times with 0.1 M PBS (pH = 7.20). The cell pellets were resuspended and 10 μM H_2_DCFDA was added; then, the cells were incubated for 30 min in the dark. Subsequently, the cells were washed three times with PBS, and the cell pellets were suspended with a final biomass of 1–5 × 10^5^ cells/ml to measure the fluorescence. Untreated yeast and H_2_O_2_ (10 μL, 30%) served as the control and positive control, respectively. The measurement results of the treatment groups were expressed as the relative ROS values compared with the control group.

### Mechanical damage determination

To detect apoptotic cell death, an Annexin V-FITC/PI double dye kit (Nanjing Jiancheng Bioengineering Institute, China) was used. The results are exhibited in Fig. S4. Due to the cell wall protection, PI (propidium iodide) dye was used to evaluate cell membrane permeability. The PI was dissolved into 0.1 M PBS (pH = 7.20) to a final concentration of 50 μg/mL and added to the yeast with a final biomass of 1–5 × 10^5^ cells/ml. Untreated yeast and H_2_O_2_ (10 μL, 30%) served as the control and positive control, respectively. High speed sorting flow cytometer (FACSAria^TM^ III, DB Company, USA) was used to analyze the fluorescence of each sample. Additionally, the morphology of wild type yeast exposed to 10 mg/L of ZnO NPs with the same conditions described for the cytotoxicity tests was inspected by TEM.

### Toxicity difference and toxicity enhancement

Herein, the toxicity difference (*T*_d_) was defined as the difference in the EC_50_ between the ZnO NPs and bulk ZnO for the yeast and its mutants. The calculation was performed using equation 3. Notably, the increasing toxicity of the particles decreased the EC_50_.


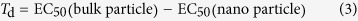


Toxicity enhancement (*T*_e_^particle^) was proposed and defined as the excess toxicity of NPs resulting from their nano-specific toxicity compared with the bulk counterpart. *T*_e_^particle^ was a dimensionless toxicity ratio calculated by the toxicity of the bulk ZnO over the toxicity of the ZnO NPs (see equation 4).


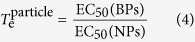


*T*_e_^ion^ was used to evaluate whether the toxic mechanism was dependent on metal ions. The comparison of ion toxicity (EC_50_ (ion)) and NPs (EC_50_ (NPs)) yielded *T*_e_^ion^ in terms of


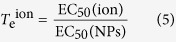


### Statistical analysis

All quantitative data were shown as the mean ± SD of a representative from at least three independent experiments. Triplicate tests were performed for each experiment. The *t* test was used to compare the differences between two groups. P < 0.05 (*) and P < 0.01 (**) were considered statistically significant and extremely statistically significant, respectively.

## Additional Information

**How to cite this article**: Zhang, W. *et al*. The neglected nano-specific toxicity of ZnO nanoparticles in the yeast *Saccharomyces cerevisiae. Sci. Rep.* **6**, 24839; doi: 10.1038/srep24839 (2016).

## Supplementary Material

Supplementary Information

## Figures and Tables

**Table 1:**
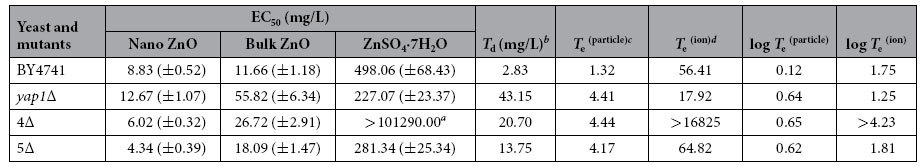
Toxicity of nano ZnO, bulk ZnO and ZnSO_4_·7H_2_O for the yeast and three mutants based on 50% cell viability with the decreasing EC_50_.

^a^EC_50_ of ZnSO_4_·7H_2_O to 4Δ was higher than 10129.00 mg/L due to the high concentration of zinc salt that induced 6.54 (±4.35) % cell death.

^b^*T*_d indicates_ toxicity differences between nano ZnO and bulk ZnO calculated through equation 3.

^c,^

^d^*T*_e_^(particle)^ and *T*_e_^(ion)^ denote the toxic ratio of bulk ZnO or zinc ion toxicity to ZnO NP toxicity calculated by equations 4 and 5, respectively.

**Table 2:** Relative toxicity contribution of particles and released ions from nano or bulk ZnO to the nominal EC_50_ in the yeast and three mutants.

yeast strains	**Particle contribution (%) from**		**Released ion contribution (%) from**	
**Nano ZnO**	**Bulk ZnO**	**Nano ZnO**	**Bulk ZnO**	
**BY4741**	93.36	92.56	6.64	7.44
***yap1*****Δ**	95.72	95.88	4.28	4.12
**4Δ**	96.68	95.42	3.32	4.58
**5Δ**	95.98	97.07	4.02	2.93

## References

[b1] Mortimer M., Kasemets K. & Kahru A. Toxicity of ZnO and CuO nanoparticles to ciliated protozoa Tetrahymena thermophila. Toxicology 269, 182–189 (2010). 19622384 10.1016/j.tox.2009.07.007

[b2] Kasemets K., Ivask A., Dubourguier H.-C. & Kahru A. Toxicity of nanoparticles of ZnO, CuO and TiO2 to yeast Saccharomyces cerevisiae. Toxicology in Vitro 23, 1116–1122 (2009). 19486936 10.1016/j.tiv.2009.05.015

[b3] Heinlaan M., Ivask A., Blinova I., Dubourguier H.-C. & Kahru A. Toxicity of nanosized and bulk ZnO, CuO and TiO2 to bacteria Vibrio fischeri and crustaceans Daphnia magna and Thamnocephalus platyurus. Chemosphere 71, 1308–1316 (2008). 18194809 10.1016/j.chemosphere.2007.11.047

[b4] George S. . Use of a High-Throughput Screening Approach Coupled with *In Vivo* Zebrafish Embryo Screening To Develop Hazard Ranking for Engineered Nanomaterials. ACS Nano 5, 1805–1817 (2011). 21323332 10.1021/nn102734s PMC3896549

[b5] Ivask A. . Mechanisms of toxic action of Ag, ZnO and CuO nanoparticles to selected ecotoxicological test organisms and mammalian cells *in vitro*: A comparative review. Nanotoxicology 8, 57–71 (2014). 24256211 10.3109/17435390.2013.855831

[b6] Ma H., Williams P. L. & Diamond S. A. Ecotoxicity of manufactured ZnO nanoparticles – A review. Environ. Pollut. 172, 76–85 (2013). 22995930 10.1016/j.envpol.2012.08.011

[b7] Xiong D., Fang T., Yu L., Sima X. & Zhu W. Effects of nano-scale TiO_2_, ZnO and their bulk counterparts on zebrafish: Acute toxicity, oxidative stress and oxidative damage. Sci. Total Environ. 409, 1444–1452 (2011). 21296382 10.1016/j.scitotenv.2011.01.015

[b8] Aruoja V., Dubourguier H.-C., Kasemets K. & Kahru A. Toxicity of nanoparticles of CuO, ZnO and TiO2 to microalgae Pseudokirchneriella subcapitata. Sci. Total Environ. 407, 1461–1468 (2009). 19038417 10.1016/j.scitotenv.2008.10.053

[b9] Yu L.-p., Fang T., Xiong D.-w., Zhu W.-t. & Sima X.-f. Comparative toxicity of nano-ZnO and bulk ZnO suspensions to zebrafish and the effects of sedimentation, OH production and particle dissolution in distilled water. J. Environ. Monit 13, 1975–1982 (2011). 21611643 10.1039/c1em10197h

[b10] Li Y., Niu J., Zhang W., Zhang L. & Shang E. Influence of Aqueous Media on the ROS-Mediated Toxicity of ZnO Nanoparticles toward Green Fluorescent Protein-Expressing Escherichia coli under UV-365 Irradiation. Langmuir 30, 2852–2862 (2014). 24568235 10.1021/la5000028

[b11] Franklin N. M. . Comparative Toxicity of Nanoparticulate ZnO, Bulk ZnO, and ZnCl2 to a Freshwater Microalga (Pseudokirchneriella subcapitata): The Importance of Particle Solubility. Environ. Sci. Technol. 41, 8484–8490 (2007). 18200883 10.1021/es071445r

[b12] Raghupathi K. R., Koodali R. T. & Manna A. C. Size-Dependent Bacterial Growth Inhibition and Mechanism of Antibacterial Activity of Zinc Oxide Nanoparticles. Langmuir 27, 4020–4028 (2011). 21401066 10.1021/la104825u

[b13] Xu M. . Challenge to assess the toxic contribution of metal cation released from nanomaterials for nanotoxicology - the case of ZnO nanoparticles. Nanoscale 5, 4763–4769 (2013). 23604040 10.1039/c3nr34251d

[b14] Xiao Y., Vijver M. G., Chen G. & Peijnenburg W. J. G. M. Toxicity and Accumulation of Cu and ZnO Nanoparticles in Daphnia magna. Environ. Sci. Technol. 49, 4657–4664 (2015). 25785366 10.1021/acs.est.5b00538

[b15] Kasemets K., Suppi S., Künnis-Beres K. & Kahru A. Toxicity of CuO Nanoparticles to Yeast Saccharomyces cerevisiae BY4741 Wild-Type and Its Nine Isogenic Single-Gene Deletion Mutants. Chem Res Toxicol 26, 356–367 (2013). 23339633 10.1021/tx300467d

[b16] Bao S., Lu Q., Fang T., Dai H. & Zhang C. An assessment of the toxicity of CuO nanoparticles using multiple-gene-deleted mutants of Saccharomyces cerevisiae. Appl. Environ. Microbiol (2015). 10.1128/AEM.02035-15 PMC4651071 26386067

[b17] Li M., Zhu L. & Lin D. Toxicity of ZnO Nanoparticles to Escherichia coli: Mechanism and the Influence of Medium Components. Environ. Sci. Technol. 45, 1977–1983 (2011). 21280647 10.1021/es102624t

[b18] Khare P. . Size dependent toxicity of zinc oxide nano-particles in soil nematode Caenorhabditis elegans. Nanotoxicology 9, 423–432 (2015). 25051332 10.3109/17435390.2014.940403

[b19] Bian S.-W., Mudunkotuwa I. A., Rupasinghe T. & Grassian V. H. Aggregation and Dissolution of 4 nm ZnO Nanoparticles in Aqueous Environments: Influence of pH, Ionic Strength, Size, and Adsorption of Humic Acid. Langmuir 27, 6059–6068 (2011). 21500814 10.1021/la200570n

[b20] Zhang M. . Creation of a Hyperpermeable Yeast Strain to Genotoxic Agents through Combined Inactivation of PDR and CWP Genes. Toxicol. Sci. 113, 401–411 (2010). 19884123 10.1093/toxsci/kfp267

[b21] Jo W. J. . Identification of Genes Involved in the Toxic Response of Saccharomyces cerevisiae against Iron and Copper Overload by Parallel Analysis of Deletion Mutants. Toxicol. Sci. 101, 140–151 (2008). 17785683 10.1093/toxsci/kfm226

[b22] Brunner T. J. . *In Vitro* Cytotoxicity of Oxide Nanoparticles: Comparison to Asbestos, Silica, and the Effect of Particle Solubility^†^. Environ. Sci. Technol. 40, 4374–4381 (2006). 16903273 10.1021/es052069i

[b23] Xia T. . Comparison of the Mechanism of Toxicity of Zinc Oxide and Cerium Oxide Nanoparticles Based on Dissolution and Oxidative Stress Properties. ACS Nano 2, 2121–2134 (2008). 19206459 10.1021/nn800511k PMC3959800

[b24] Adam N. . The chronic toxicity of ZnO nanoparticles and ZnCl2 to Daphnia magna and the use of different methods to assess nanoparticle aggregation and dissolution. Nanotoxicology 8, 709–717 (2014). 23837602 10.3109/17435390.2013.822594

[b25] Xia T. . Comparison of the Abilities of Ambient and Manufactured Nanoparticles To Induce Cellular Toxicity According to an Oxidative Stress Paradigm. Nano Lett 6, 1794–1807 (2006). 16895376 10.1021/nl061025k

[b26] Battin T. J., Kammer F. v. d., Weilhartner A., Ottofuelling S. & Hofmann T. Nanostructured TiO2: Transport Behavior and Effects on Aquatic Microbial Communities under Environmental Conditions. Environ. Sci. Technol. 43, 8098–8104 (2009). 19924929 10.1021/es9017046

[b27] Thill A. . Cytotoxicity of CeO2 Nanoparticles for Escherichia coli. Physico-Chemical Insight of the Cytotoxicity Mechanism. Environ. Sci. Technol. 40, 6151–6156 (2006). 17051814 10.1021/es060999b

[b28] Zhang L., Jiang Y., Ding Y., Povey M. & York D. Investigation into the antibacterial behaviour of suspensions of ZnO nanoparticles (ZnO nanofluids). J Nanopart Res 9, 479–489 (2007).

[b29] Wahab R., Mishra A., Yun S.-I., Kim Y.-S. & Shin H.-S. Antibacterial activity of ZnO nanoparticles prepared via non-hydrolytic solution route. Appl Microbiol Biotechnol 87, 1917–1925 (2010). 20526594 10.1007/s00253-010-2692-2

[b30] Brayner R. . Toxicological Impact Studies Based on Escherichia coli Bacteria in Ultrafine ZnO Nanoparticles Colloidal Medium. Nano Lett 6, 866–870 (2006). 16608300 10.1021/nl052326h

[b31] Huang Z. . Toxicological Effect of ZnO Nanoparticles Based on Bacteria. Langmuir 24, 4140–4144 (2008). 18341364 10.1021/la7035949

[b32] Bacchetta R. . Evidence and uptake routes for Zinc oxide nanoparticles through the gastrointestinal barrier in Xenopus laevis. Nanotoxicology 8, 728–744, (2014). 23848496 10.3109/17435390.2013.824128

[b33] Hao L., Chen L., Hao J. & Zhong N. Bioaccumulation and sub-acute toxicity of zinc oxide nanoparticles in juvenile carp (Cyprinus carpio): A comparative study with its bulk counterparts. Ecotoxicol. Environ. Saf. 91, 52–60 (2013). 23375439 10.1016/j.ecoenv.2013.01.007

[b34] Sabella S. . A general mechanism for intracellular toxicity of metal-containing nanoparticles. Nanoscale 6, 7052–7061 (2014). 24842463 10.1039/c4nr01234h PMC4120234

[b35] Hsiao I. L., Hsieh Y.-K., Wang C.-F., Chen I. C. & Huang Y.-J. Trojan-Horse Mechanism in the Cellular Uptake of Silver Nanoparticles Verified by Direct Intra- and Extracellular Silver Speciation Analysis. Environ. Sci. Technol. 49, 3813–3821 (2015). 25692749 10.1021/es504705p

[b36] Zhang L. . Mechanistic investigation into antibacterial behaviour of suspensions of ZnO nanoparticles against E. coli. J Nanopart Res 12, 1625–1636 (2010).

[b37] Schramm F., Müller A., Hammer H., Paschke A. & Schüürmann G. Epoxide and Thiirane Toxicity *In vitro* with the Ciliates Tetrahymena pyriformis: Structural Alerts Indicating Excess Toxicity. Environ. Sci. Technol. 45, 5812–5819 (2011). 21662985 10.1021/es200081n

[b38] Navarro E. . Toxicity of Silver Nanoparticles to Chlamydomonas reinhardtii. Environ. Sci. Technol. 42, 8959–8964 (2008). 19192825 10.1021/es801785m

[b39] Levard C. . Effect of Chloride on the Dissolution Rate of Silver Nanoparticles and Toxicity to E. coli. Environ. Sci. Technol. 47, 5738–5745 (2013). 23641814 10.1021/es400396f

[b40] Shoults-Wilson W. A. . Effect of silver nanoparticle surface coating on bioaccumulation and reproductive toxicity in earthworms (Eisenia fetida). Nanotoxicology 5, 432–444 (2011). 21142839 10.3109/17435390.2010.537382

[b41] Påhlsson A.-M. Toxicity of heavy metals (Zn, Cu, Cd, Pb) to vascular plants. Water Air Soil Pollut 47, 287–319 (1989).

[b42] Hallman P., Perrin D. & Watt A. E. The computed distribution of copper (II) and zinc (II) ions among seventeen amino acids present in human blood plasma. Biochem. J 121, 549–555 (1971). 5119792 10.1042/bj1210549 PMC1176604

