# Supplementary material for: The neglected nano-specific toxicity of ZnO nanoparticles in the yeast Saccharomyces cerevisiae
Source: Sci Rep. 2016 Apr 20;6:24839. doi: 10.1038/srep24839 (PMC4837412; doi:10.1038/srep24839)
Supplement: Supplementary Information [file srep24839-s1.pdf]

Supporting information

The neglected nano-specific toxicity of ZnO nanoparticles in the yeast *Saccharomyces cerevisiae*

Weicheng Zhang,[a] Shaopan Bao,[a,b] and Tao Fang*[a]

Table S1 The information provided for ZnO NPs' size and genotype of yeast strains, zeta potential and hydrodynamic diameter of ZnO particles of at 1 and 6 h time points.

Particles	Size (nm)[b]	Hydrodynamic diameter[c] (nm)		Zeta potential[c] (eV)	
		1 h	6 h	1 h	6 h
Nano ZnO (30 mg/L)	20	790.533 (±157.752)	1883.000 (±64.967)	25.033 (±0.306)	23.400 (±0.173)
Nano ZnO (300 mg/L)	20	443.512 (±24.962)	601.312 (±34.837)	23.133 (±0.814)	25.033 (±0.569)
Bulk ZnO (30 mg/L)		727.667 (±19.072)	723.733 (±23.599)	–18.067 (±0.404)	–17.833 (±0.751)
Bulk ZnO (300 mg/L)		762.934 (±11.314)	1034.667 (±16.660)	–11.733 (±0.643)	–9.933 (±0. 513)
**Strains[a]**	**Genotype**				
Wild type (BY4741)	**MATa** *his3Δ0 leu2Δ0 met15Δ0 ura3Δ0*			–40.433 (±0.569)	–28.5 (±3.111)
yap1 mutant (*yap1Δ*)	**MATa** *his3Δ0 leu2Δ0 met15Δ0 ura3Δ0 yap1Δ::hisG*			–34.400 (±1.587)	–30.15 (±0.778)
Quadruple mutant (4Δ)	**MATa** *his3Δ0 leu2Δ0 met15Δ0 ura3Δ0 snq2Δ::KanMX, pdr5Δ::LEU2, cwp1Δ::hisG,cwp2Δ::HIS3*			–30.200 (±2.982)	–25.2 (±0.424)
Quintuple mutant (5Δ)	**MATa** *his3Δ0 leu2Δ0 met15Δ0 ura3Δ0 snq2Δ::KanMX, pdr5Δ::LEU2, cwp1Δ::hisG, cwp2Δ::HIS3, yap1Δ::hisG*			–30.500 (±2.458)	–25.7 (±0.624)

[a]the applied yeast strains in present research were donated by lab of aquatic animal protein engineering, Institute of Hydrobiology, Chinese Academy of Sciences. [b]particles size of ZnO NPs was from Beijing Nachen S&T Ltd. [c]Both of hydrodiameter and zeta potential for nano and bulk ZnO at two time points expressed as average values with standard deviation from 6 replications.

[a] Institute of Hydrobiology, Chinese Academy of Sciences, Wuhan, 430072, China. Email: fangt@ihb.ac.cn
[b] Graduate University of Chinese Academy of Sciences, Beijing 100049, China

Table S2. Toxicity data for ZnO NPs and CuO NPs as well as their bulk counterpart and salt to different organisms were from literatures.

Organism	Exposure duration	NPs size (nm)		E(L)C$_{50}$ mg/L			E(L)C$_{50}$ mg/L			T_e ^{particle}		T_e ^{ion}		log T_e ^{particle}		log T_e ^{ion}	
		ZnO	CuO	n ZnO	b ZnO	$ZnSO_4$ •$7H_2O$	n CuO	b CuO	$CuSO_4$	ZnO	CuO	ZnO	CuO	ZnO	CuO	ZnO	CuO
Saccharomyces cerevisiae[1]	8 h	50-70	30	121	134	331	21.6	2031	28.6	1.11	94.03	2.74	1.32	0.05	1.97	0.44	0.12
Vibrio fischeri[2]	0.5 h	50-70	30	1.9	1.8	1.1	79	3811	1.6	0.95	48.24	0.58	0.02	–0.02	1.68	–0.24	–1.70
Daphnia magna[2]	48 h	50-70	30	3.2	8.8	6.1	3.2	164.8	0.17	2.75	51.50	1.91	0.05	0.44	1.71	0.28	–1.30
Thamnocephalus platyurus[2]	24 h	50-70	30	0.18	0.24	0.98	2.1	94.5	0.11	1.33	45.00	5.44	0.05	0.12	1.65	0.74	–1.30
Tetraphymena thermophile[3]	24 h	50-70	30	6.8	7.4	6.7	97.9	1966	1.4	1.09	20.08	0.99	0.014	0.04	1.30	0.00	-1.85
Pseudokirchneriella subcapitata[4]	96 h	50-70	30	0.042	0.037	0.042	0.71	11.55	0.02	0.88	16.27	1.00	0.03	–0.06	1.21	0.00	–1.52

T_e ^{particle} and T_e ^{particle} were calculated through equation 4 and 5.

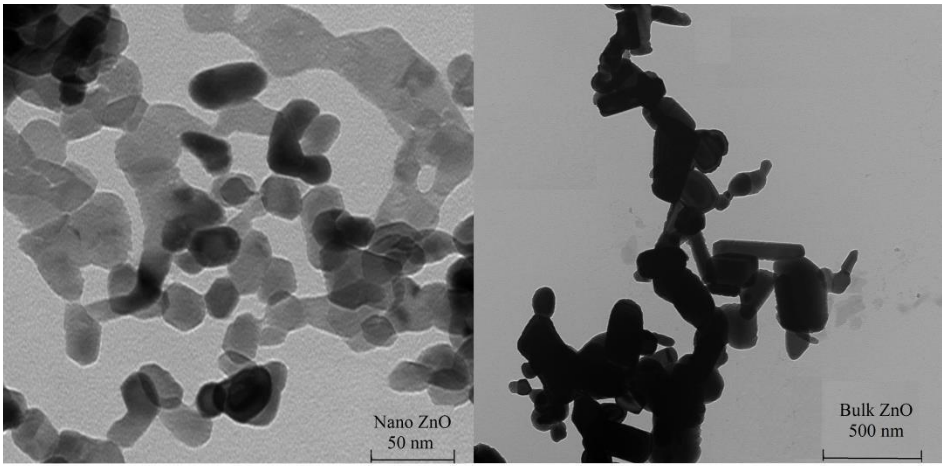

Figure S1 TEM images of nano and bulk ZnO (10 mg/L) in Deionized-water.

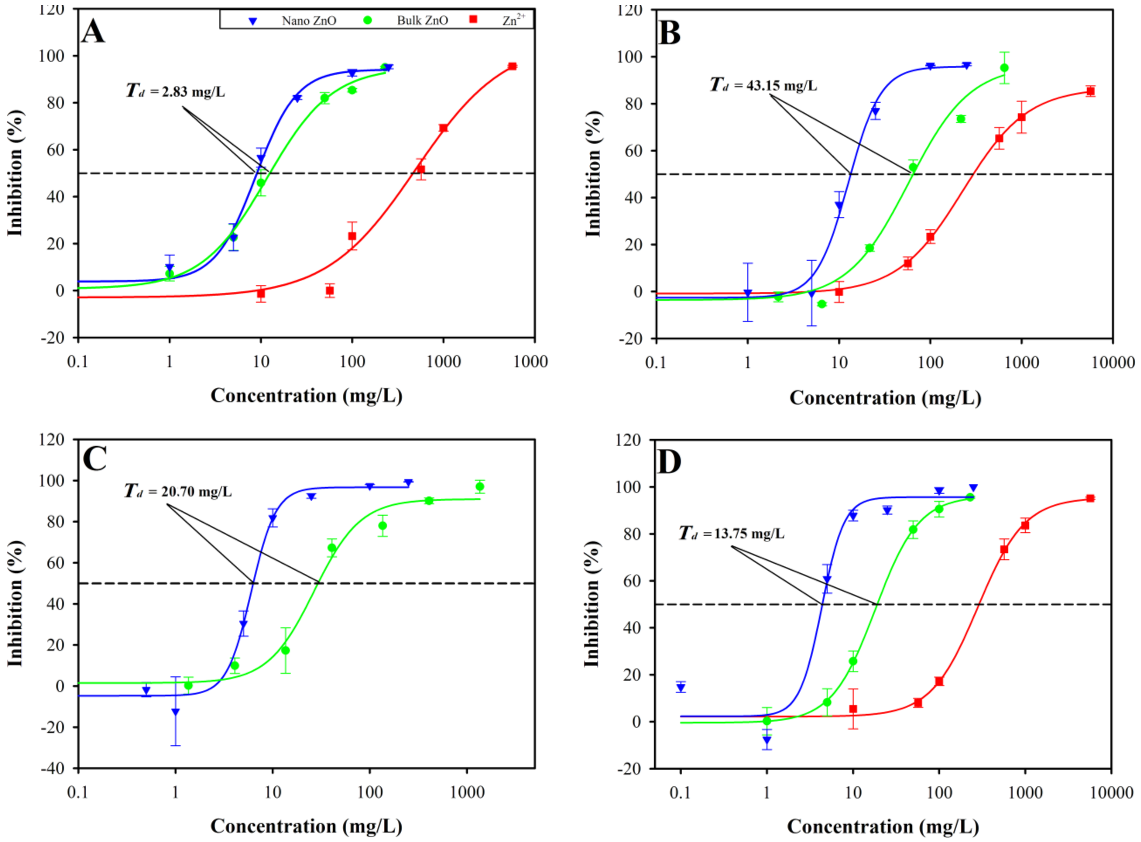

Figure S2 The cytotoxicity of ZnO NPs, bulk ZnO and zinc salt to yeast strains. A: BY4741; B: *yap1*Δ; C: 4Δ; D: 5Δ. T_d for each strain was calculated by equation 3.

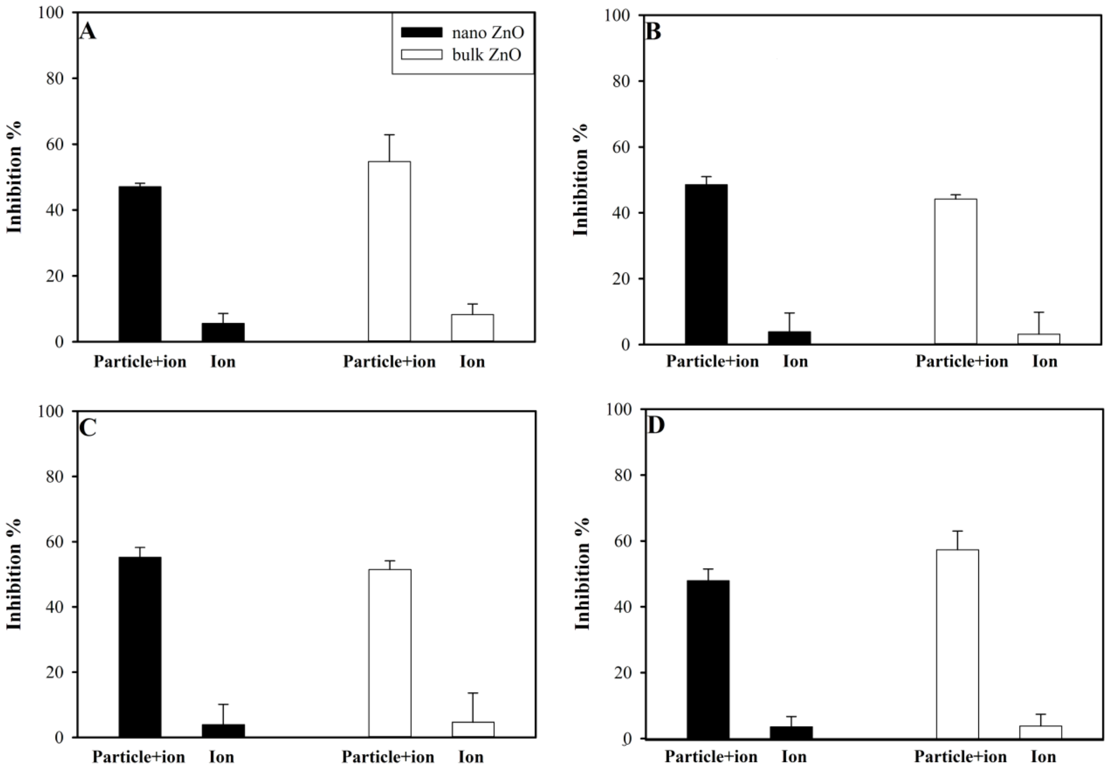

Figure S3 Inhibition caused by calculated EC50 of ZnO (nano and bulk) particles and its correspondingly released Zn^{2+} to yeast and mutants. A: BY4741; B: *yap1*Δ; C: 4Δ; D: 5Δ. n = 3, from three independent experiments.

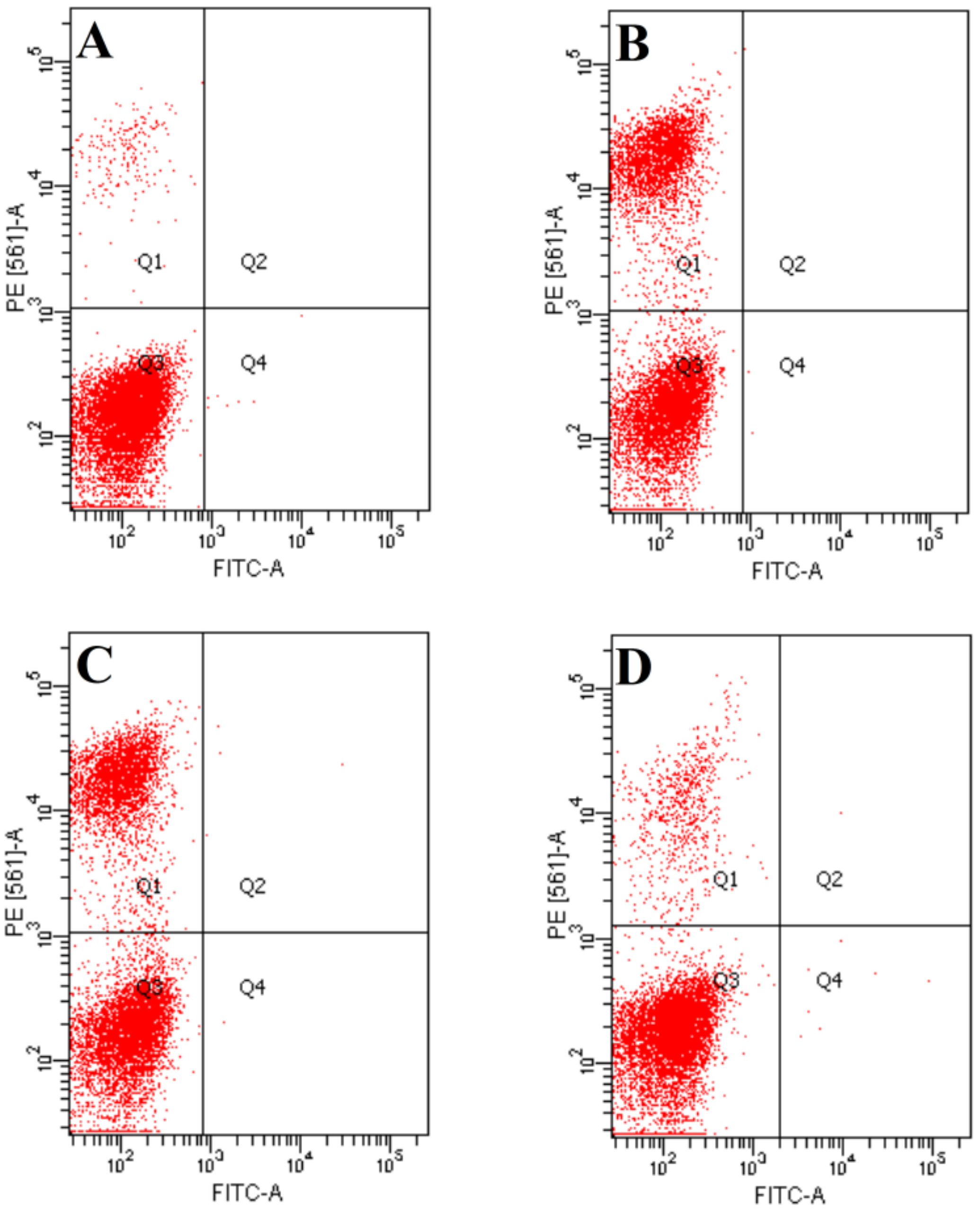

Figure S4 The apoptosis experiments were conducted using double dye Annexin V-FITC/ PI to wild type yeast. A: Blank (0 mg/L of ZnO NPs), B: Treatment 1 (5 mg/L of ZnO NPs), C: Treatment 2 (5 mg/L of bulk ZnO), D: Treatment 4 (5 mg/L of Zn^{2+}). Q1 region denotes the cell dyed by PI, Q4 region denotes the cell dyed by Annexin V-FITC, and Q2 region denotes the cell dyed by both Annexin V-FITC and PI.

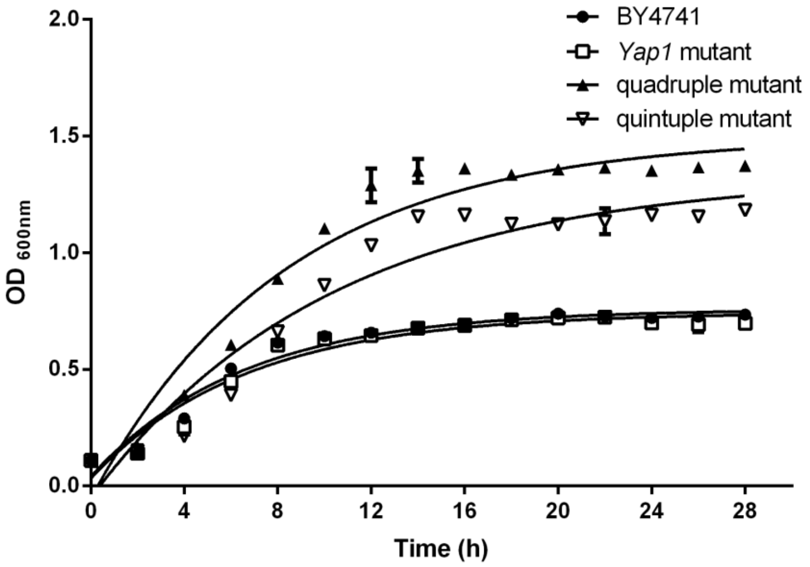

Figure S5 The Yeast *Saccharomyces cerevisiae* wild-type BY4741 and mutants' growth in SD medium at 30 ℃ with 200 rpm continuous shaking during 28 h.[5]

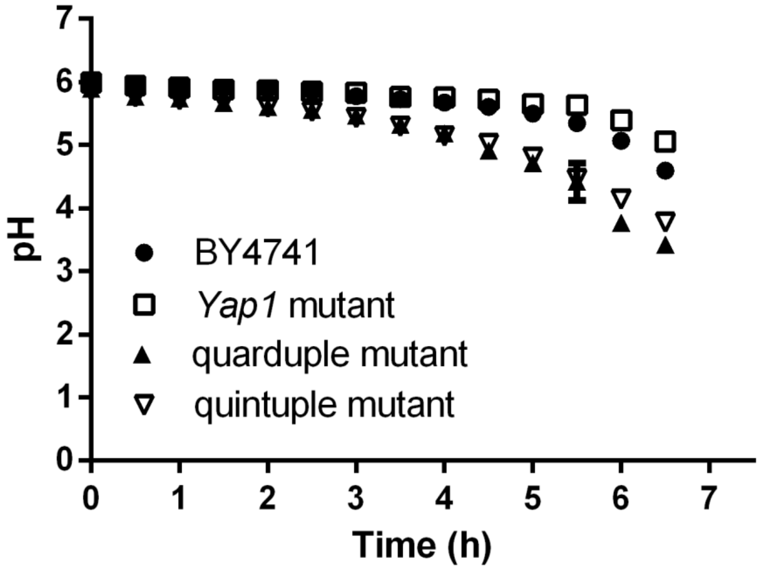

Figure S5 Dynamic variation of pH values in the SD medium over time in the presences of yeast wild-type and mutants. Data are means ±standard error of 3 independent replicates.[5]

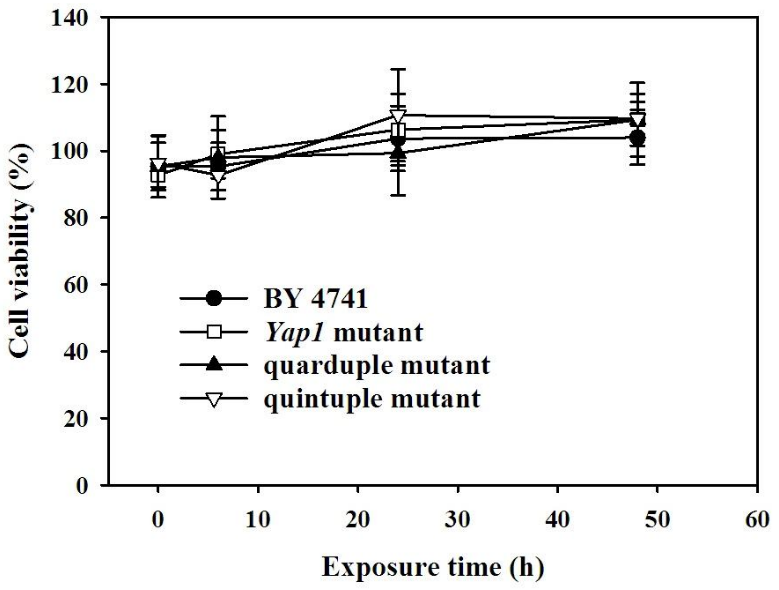

Figure S7 The cell viability of yeast strains under non-stress condition at different exposure time. Data are means ± standard error of 3 independent replicates.The increased cell viability (> 100%) at 24 and 48 h could be explained by the volatilization of deionized water during the exposure duration.

Reference:

1. Kasemets, K.; Ivask, A.; Dubourguier, H.-C.; Kahru, A., Toxicity of nanoparticles of ZnO, CuO and TiO2 to yeast Saccharomyces cerevisiae. *Toxicology in Vitro* **2009,** *23* (6), 1116-1122.
2. Heinlaan, M.; Ivask, A.; Blinova, I.; Dubourguier, H.-C.; Kahru, A., Toxicity of nanosized and bulk ZnO, CuO and TiO2 to bacteria Vibrio fischeri and crustaceans Daphnia magna and Thamnocephalus platyurus. *Chemosphere* **2008,** *71* (7), 1308-1316.
3. Mortimer, M.; Kasemets, K.; Kahru, A., Toxicity of ZnO and CuO nanoparticles to ciliated protozoa Tetrahymena thermophila. *Toxicology* **2010,** *269* (2–3), 182-189.
4. Aruoja, V.; Dubourguier, H.-C.; Kasemets, K.; Kahru, A., Toxicity of nanoparticles of CuO, ZnO and TiO2 to microalgae Pseudokirchneriella subcapitata. *Science of The Total Environment* **2009,** *407* (4), 1461-1468.
5. Bao, S.; Lu, Q.; Fang, T.; Dai, H.; Zhang, C., An assessment of the toxicity of CuO nanoparticles using multiple-gene-deleted mutants of Saccharomyces cerevisiae. *Applied and Environmental Microbiology* **2015**.
